# Necrotizing Fasciitis in a Child in a Third-World Country: A Case Report

**DOI:** 10.7759/cureus.101927

**Published:** 2026-01-20

**Authors:** Francisco J Quintana, Soshana S Hunter

**Affiliations:** 1 Plastic and Reconstructive Surgery, Hospital Militar Escuela Dr. Alejandro Dávila Bolaños, Managua, NIC; 2 Surgery, Hospital Antonio Lenin Fonseca, Managua, NIC; 3 School of Medicine, Universidad Americana (UAM), Managua, NIC

**Keywords:** necrotizing fasciitis, plastic surgery procedures, reconstructive surgery, skin graft, skin infection

## Abstract

Necrotizing fasciitis is a soft tissue infection that represents an emergency due to its alarmingly fast progression. It requires surgical debridement and antibiotics, but more advanced interventions can be needed, such as reconstructive procedures, including an Ollier-Thiersch dermograft, as in the case we are presenting.

We introduce the history and clinical evolution of a patient with necrotizing fasciitis, who underwent dermoplasty of the right temporal region.

This case report was conducted in the Plastic Surgery Service at Hospital Militar Escuela Dr. Alejandro Dávila Bolaños, during 2025. Demographic data, clinical course, laboratory studies, and the therapeutic approach were documented. Informed consent was obtained for publication of the case.

A one-year-old male patient presented with punctate lesions in the periorbital region. On physical examination, edema and erythema were observed, which progressed to abscess and necrosis. Antibiotic therapy, wound irrigation, and debridement were initiated, followed by wound closure using Ollier-Thiersch dermoplasty. The patient had a satisfactory clinical outcome.

We present the clinical evolution and surgical approach of a patient with necrotizing fasciitis, who underwent debridement, negative pressure wound therapy, and dermoplasty.

## Introduction

Necrotizing fasciitis is a rare and rapidly progressive skin and soft tissue infection that results in fascia and subcutaneous tissue necrosis; therefore, timely diagnosis and appropriate management of the disease are essential for a favorable prognosis. The mean age of patients with this disease is 38-44 years, with a rare occurrence in children. Pediatric cases have been reported from resource-poor nations, such as Nicaragua, where deficient hygiene practices are prevalent [1].

The etiology of the infection can be necrotizing fasciitis type 1 (polymicrobial), caused by aerobic bacteria such as facultative streptococci, enterococci, staphylococci, gram-negative bacilli (such as *Escherichia coli*, *Klebsiella*, *Pseudomonas*, among others), and anaerobes (*Bacteroides*, *Peptostreptococcus* and *Clostridium* spp.), or necrotizing fasciitis type 2 (monomicrobial), with group A streptococcus being the most frequent microorganism [2,3].

The natural course of the disease is characterized by its onset on the superficial fascia, where bacteria proliferate and release toxins and enzymes that facilitate the horizontal spread of the infection, resulting in necrosis and thrombosis of small vessels. Once the tissue is destroyed, pathogen invasion progresses vertically, affecting both deep layers and the superficial dermis, at which point the clinical signs of the condition appear: pain and inflammation, which progress to firm edema and tenderness with increased local temperature [4,5].

Gradually, the epidermis develops dark red induration, along with fluid-filled blisters of a bluish or purplish hue; following this, the skin becomes friable and turns bluish or black [6,7]. Dissemination of the infection toward the deep fascia causes the tissue to take on a grayish-brown coloration. Sometimes, septic shock and hypotension can appear [6].

Diagnosis includes early recognition of four key clinical signs: edema and induration beyond the erythematous area, blisters or bullae, crepitation or the presence of gas on imaging studies, and the absence of associated lymphangitis and lymphadenitis, complemented by imaging studies, with magnetic resonance imaging being the most sensitive technique. However, X-ray and computed tomography can be used to confirm the presence of gas [4].

The mainstay treatment is surgical debridement of the necrotic area until viable tissue is encountered to control the infection, alongside the use of broad-spectrum antibiotic therapy. Nonetheless, the extent of the disease may complicate primary closure, which is why a reconstructive procedure, in this case, Ollier-Thiersch dermoplasty, can be necessary [8].

## Case presentation

A one-year-old boy with a history of West syndrome, gastroesophageal reflux disease, and chronic aspiration syndrome presented with punctate lesions in the periorbital region, associated with a four-day history of fever. On physical examination, edema and erythema were observed, with portals of entry located in the right temporal region.

**Figure 1:**
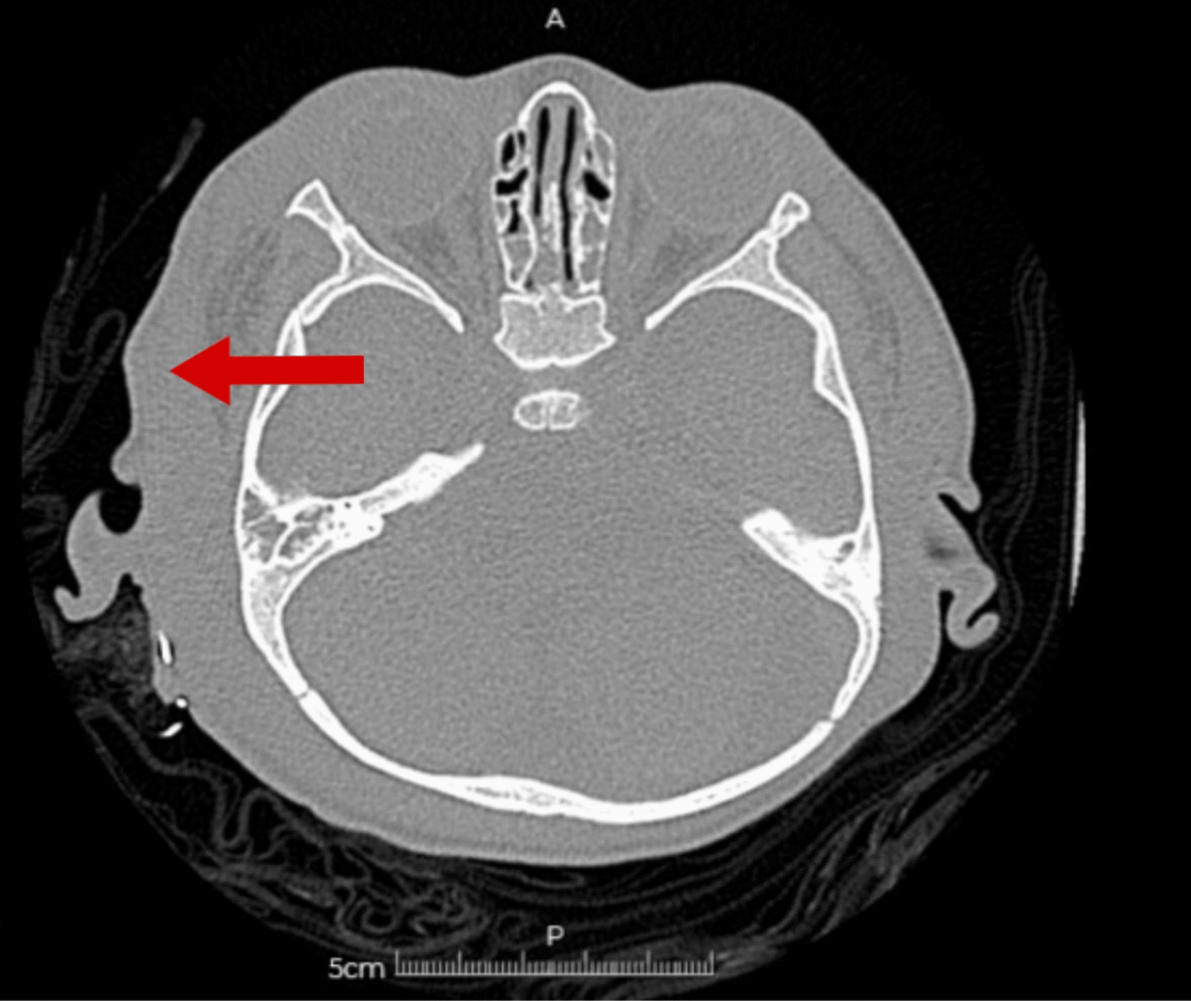
Head CT with an arrow pointing to an area of low attenuation within the subcutaneous tissue at the superolateral aspect of the right periorbital region, with irregular, poorly defined borders, associated with marked edema of the periorbital soft tissues.

A diagnosis of right periorbital cellulitis was made, and the patient was admitted to the inpatient ward to complete antibiotic treatment with a third-generation cephalosporin and lacosamide. A CT scan was indicated to evaluate compromise of other anatomical structures or complications; it suggested a right periorbital and ipsilateral retroauricular collection, accompanied by significant soft tissue edema in the affected areas (Figure 1). Blood cultures were negative, and laboratory studies revealed elevated acute-phase reactants (Table 1).

**Table 1:** Acute-phase reactants. ELCIA: Electrochemiluminescence immunoassay.

Exam	Results	Measurement unit	Reference values
Procalcitonin (ECLIA)	100	ng/mL	<0.5: low risk of sepsis; ≥0.5: high risk of sepsis

After two days, the patient was transferred to the pediatric intensive care unit due to hypoactivity, poor general condition, head nodding, and clinical signs suggestive of sepsis, along with respiratory failure. Blood gas analysis demonstrated acidosis (Table 2).

**Table 2:** Blood gas analysis. PO₂: Arterial partial pressure of oxygen; PCO₂: Arterial partial pressure of carbon dioxide; BE: Base excess.

Exam	Results	Measurement unit	Reference values
pH	7.28	-	7.35-7.45
PO₂	34.2	mmHg	80-100
PCO₂	31.5	mmHg	35-45
BE	-10.98	mmol/L	-2 to +2

**Figure 2:**
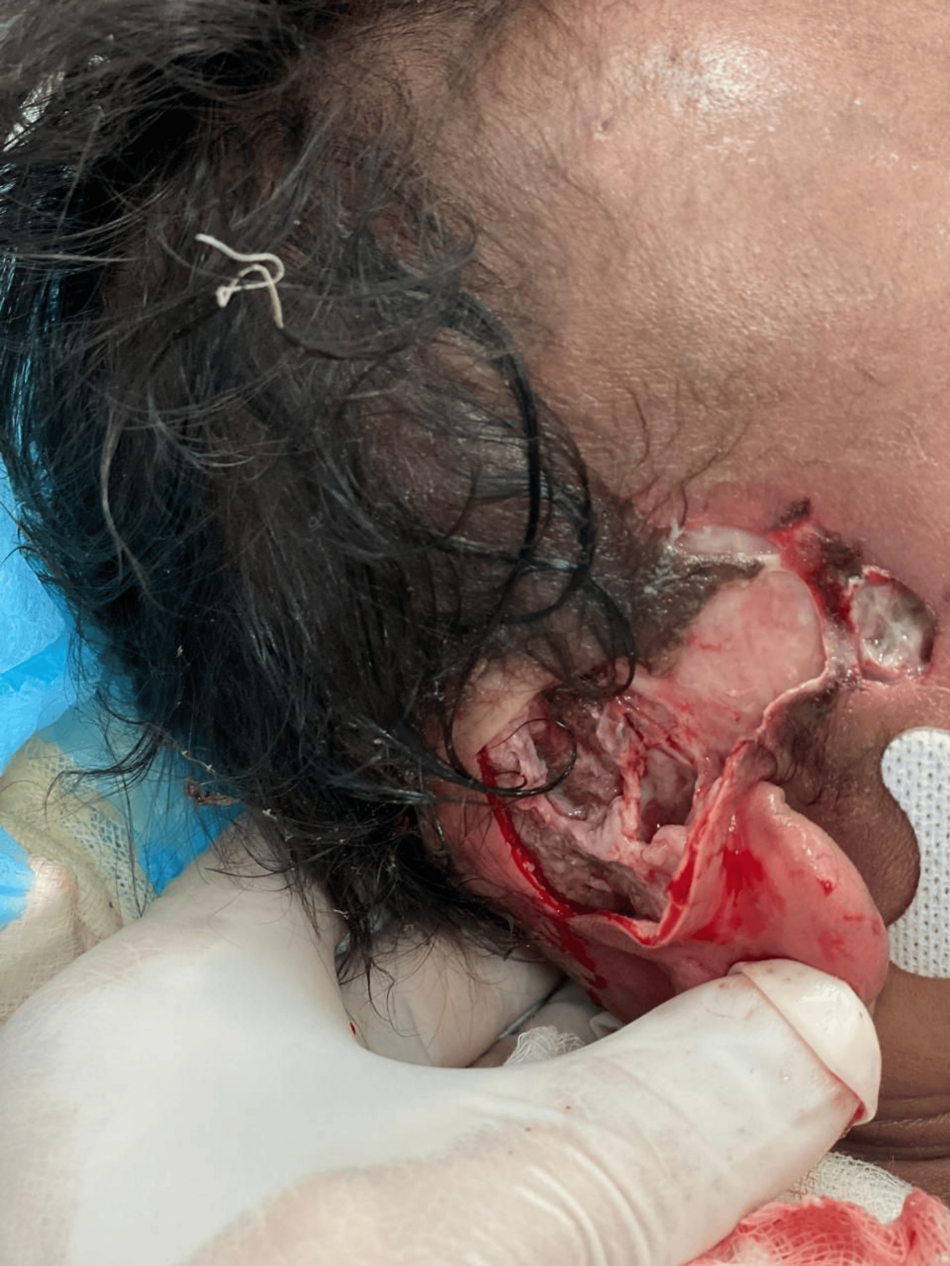
Necrotizing fasciitis involving the right temporal region.

On physical examination, a periorbital abscess with spontaneous drainage was observed, along with necrotic subcutaneous tissue not integrated with the cranial surface and exposure of the temporal muscle (Figure 2). This prompted the diagnosis of necrotizing fasciitis of polymicrobial origin, complicated by septic shock, and managed with carbapenems and glycopeptides.

**Figure 3:**
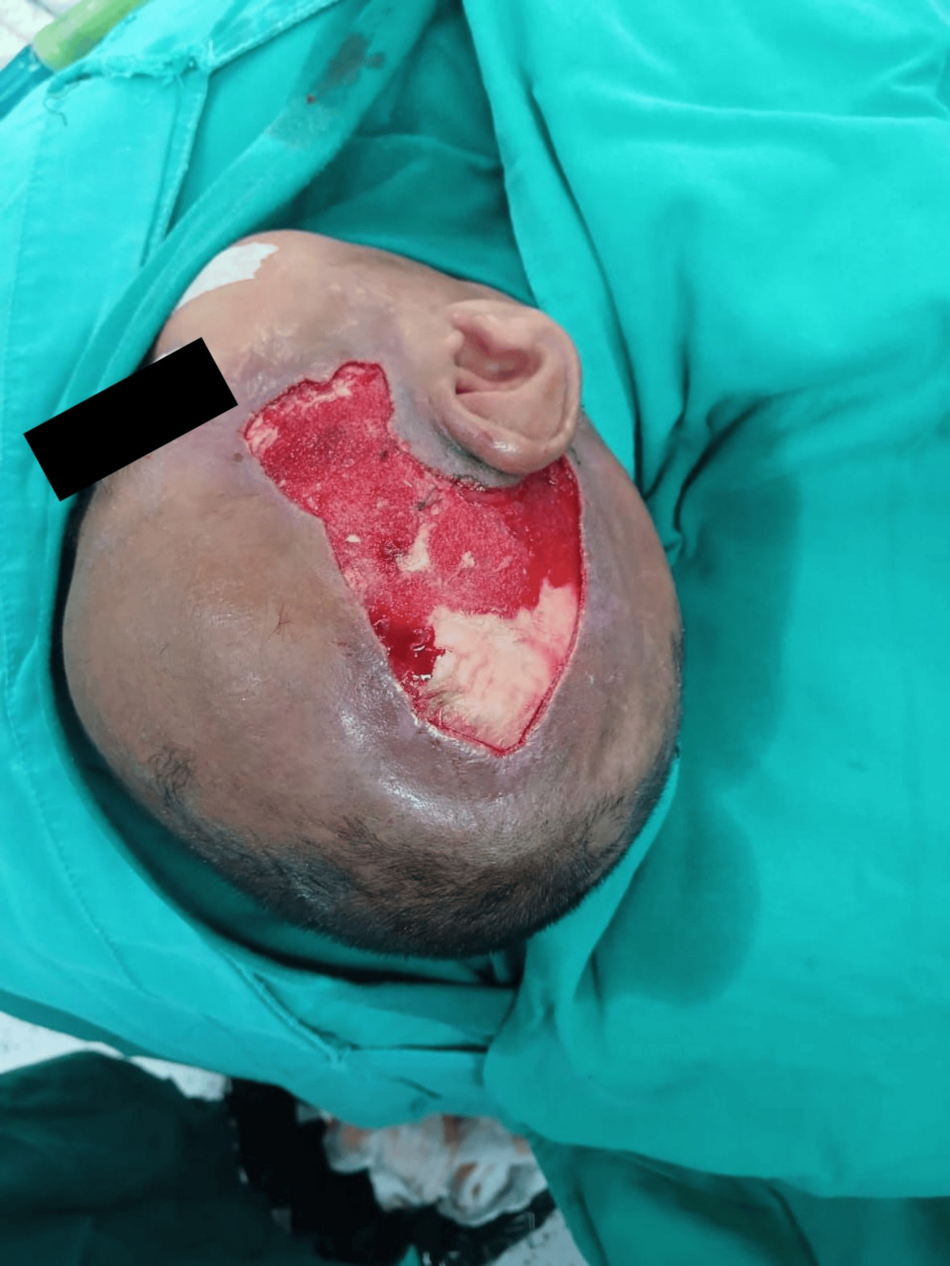
Wound following irrigation and surgical debridement.

A plastic surgery consultation was requested, and the team performed surgical irrigation, escharotomy, and debridement with drainage of the right temporal region (Figure 3), followed by placement of calcium alginate dressings. Subsequently, they validated the need for fasciotomy but did not proceed due to the high probability of death related to the inflammatory response to the intervention. 

On the seventh day of the hospital stay, another head CT was performed, which revealed right periorbital cellulitis with improvement compared with previous studies, a collection in the right parietotemporal soft tissue, bilateral mastoiditis, and pansinusitis. The ear CT demonstrated bilateral otomastoiditis, while the neck CT showed a small collection in the soft tissue of the right lower mandibular region.

**Figure 4:**
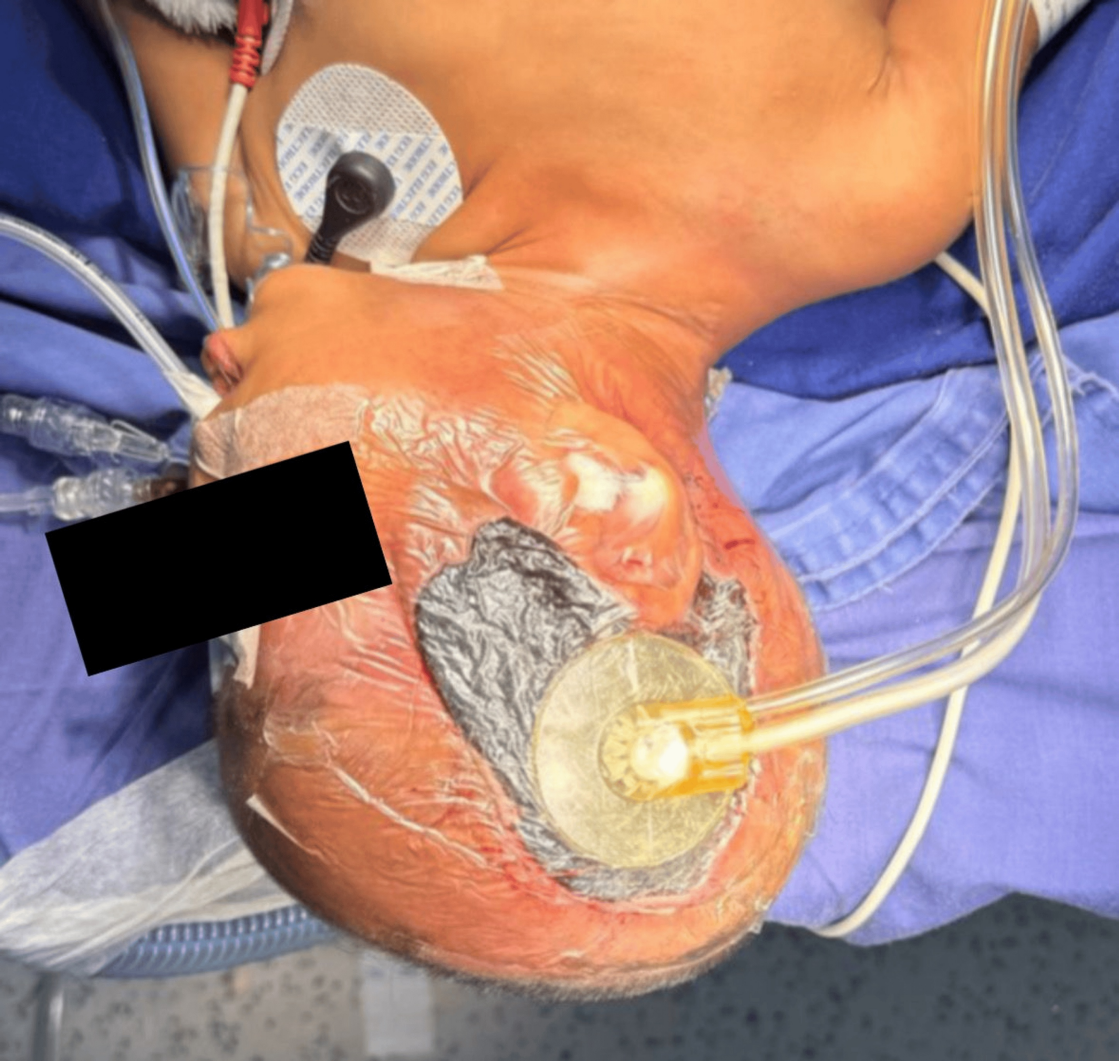
Placement of a VAC system. VAC: Vacuum-assisted closure.

Following that, two debridement procedures were performed. Five days later, he was taken to the operating room with the intention of placing a vacuum-assisted closure (VAC) system; however, the condition of the skin and tissue precluded the use of such therapy, as it would have damaged the wound edges and thereby increased its diameter. As a result, calcium alginate was used as a biological substitute dressing. After 48 hours, the patient underwent surgery for placement of a VAC system set to continuous pressure of 25 mmHg, which was successfully performed (Figure 4).

Five days later, further interventions were performed, during which the patient required surgical debridement and placement of hydrocolloid dressings. A biopsy of the temporal bone fascia was taken, revealing fibrotic tissue and extensive necrotic areas with acute and chronic inflammation, findings consistent with necrotizing fasciitis.

**Figure 5:**
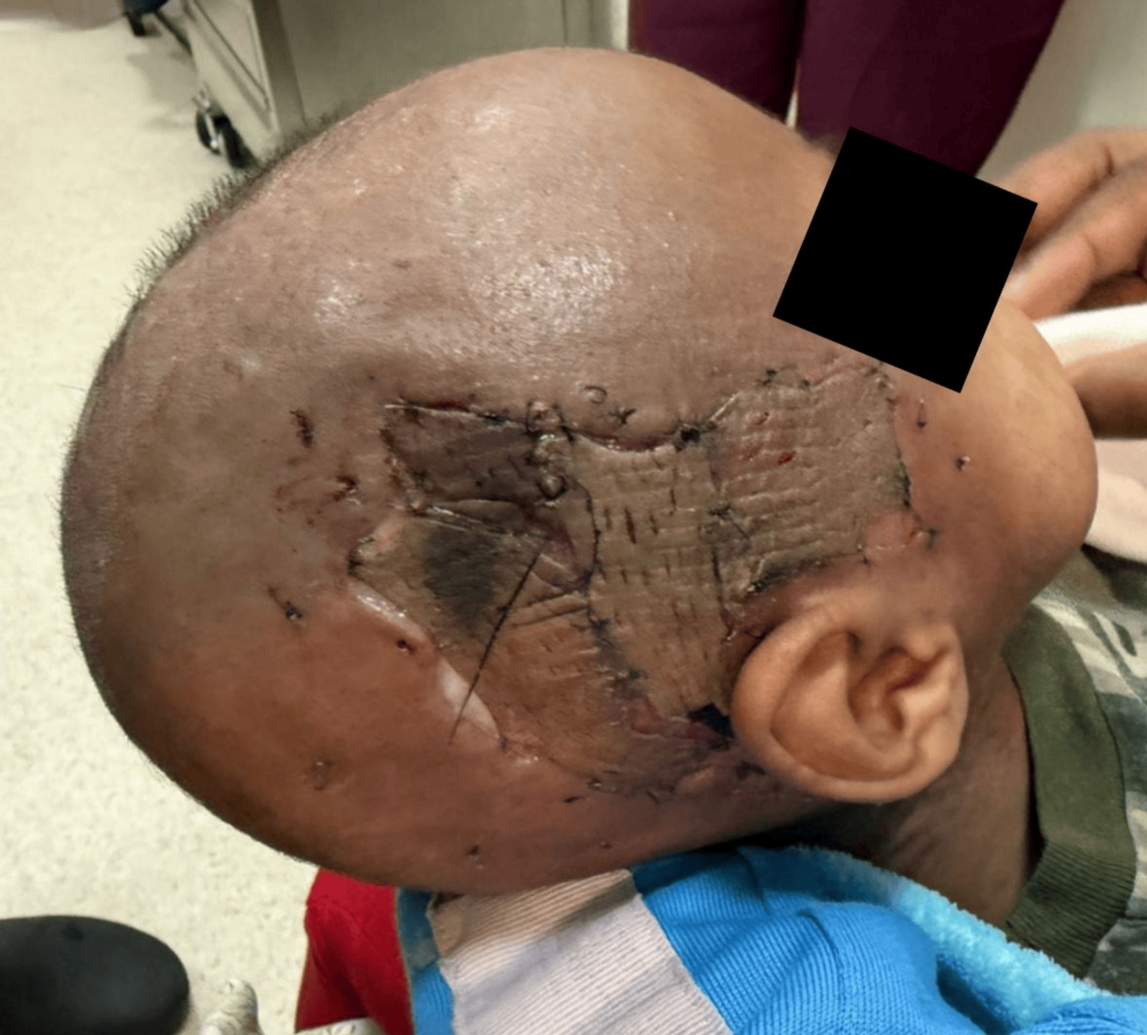
Ollier-Thiersch dermoplasty.

After two surgical procedures involving irrigation and replacement of the hydrocolloid dressings, the patient was in optimal condition to undergo dermoplasty of the affected area, which was successfully carried out without complications using a skin graft obtained from the anterior region of the right thigh (Figure 5).

**Figure 6:**
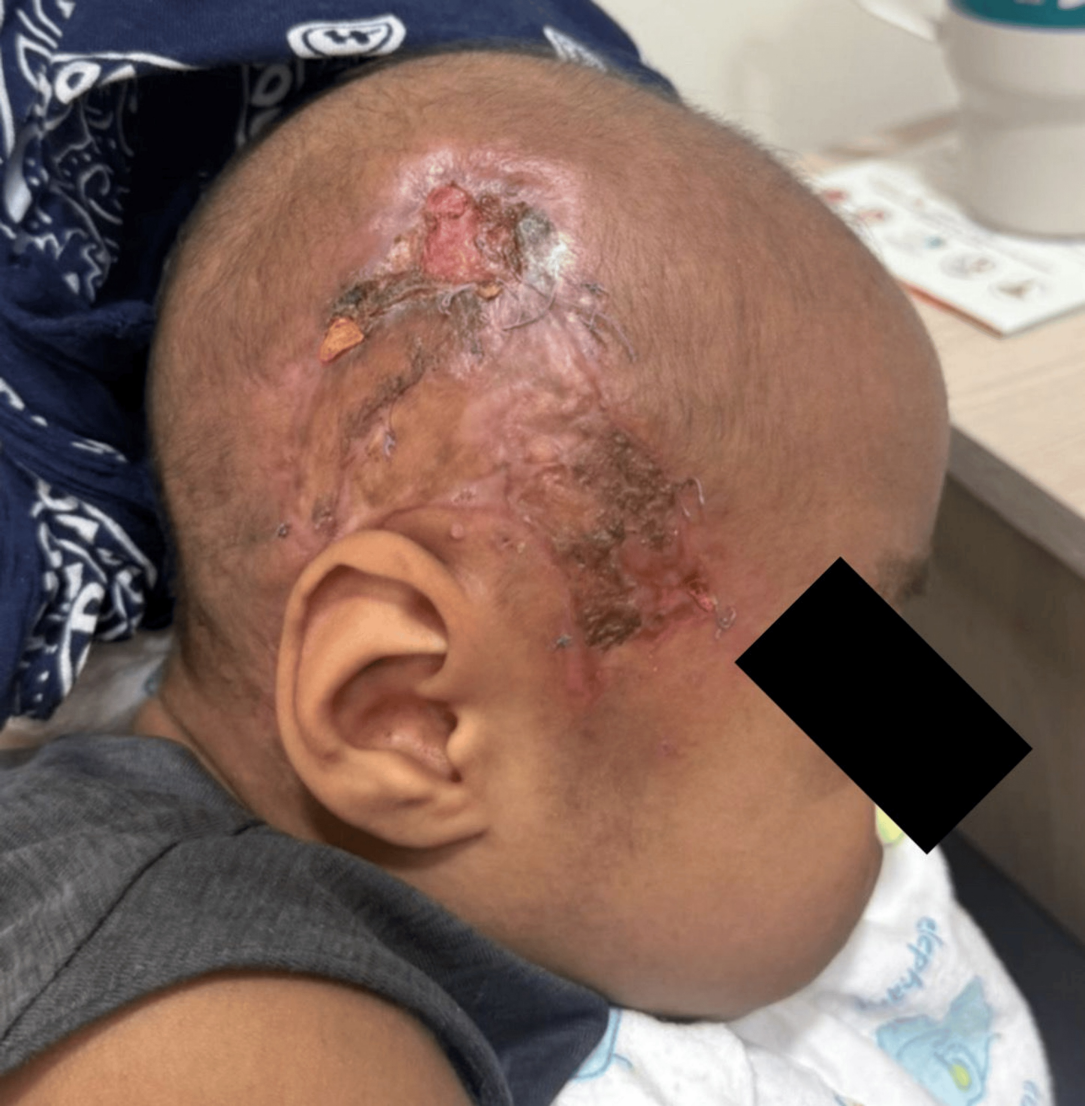
Final result of Ollier-Thiersch dermoplasty.

Finally, after 35 days of hospital stay and eight surgical interventions, the patient was discharged with general instructions and scheduled follow-up appointments for multidisciplinary management involving the appropriate subspecialties, with satisfactory outcomes (Figure 6).

## Discussion

Necrotizing fasciitis is a rapidly progressive skin and soft tissue infection that can result in sepsis, multi-organ failure, and death. It is a disease with multiple risk factors, etiologic agents, and pathophysiological mechanisms, with an extensive clinical presentation showing local manifestations such as edema, erythema, tenderness, and necrosis, as well as systemic signs including fever, tachycardia, and hypotension.

Histological examination reveals necrosis with small- and medium-vessel occlusion, with neutrophil infiltration, destruction and liquefaction of adipose tissue, and bacterial proliferation. Gram staining allows visualization of the etiologic agent [6].

Prompt and precise management is critical, as it directly influences the patient’s clinical course and prognosis. Clinical manifestations serve as the main diagnostic indicators, complemented by a thorough and detailed history and physical examination aimed at identifying a portal of entry for the infection, which is present in 80% of necrotizing fasciitis cases. Imaging studies can also be useful to determine the presence of the infectious process, noting that CT demonstrates a sensitivity of 100% and specificity of 80-98%, while MRI shows sensitivity of 90-100% and specificity of 50-80% [4,9].

Once the diagnosis is confirmed, treatment must be initiated immediately, consisting of appropriate control of the infectious source with antimicrobial therapy and surgical interventions such as irrigation and debridement.

This case illustrates the rapid progression of the disease, where the initial diagnosis of periorbital cellulitis quickly escalated to necrotizing fasciitis and sepsis within two days. The presentation started with periorbital edema that then progressed to an abscess with spontaneous drainage, necrotic tissue not adherent to the cranial surface, and exposure of the temporal muscle, with a portal of entry in the right temporal region and signs of multi-organ failure. These findings align with the literature, reinforcing the importance of a timely and accurate approach.

According to the etiologic classification, the patient presented with type 1 necrotizing fasciitis (polymicrobial); therefore, the antibiotic of choice was broad-spectrum therapy, which managed to control the infection.

The diagnosis was supported by multiple CT scans, which determined the extent and severity of the initial lesion and illustrated the pathophysiology of the disease, with both vertical and horizontal progression. The process began as a periorbital lesion and ultimately involved the ear, resulting in bilateral otomastoiditis, and the neck, with a soft tissue collection in the right lower mandibular region.

Histologically, the findings aligned with those described in the literature, showing extensive areas of necrosis, accompanied by both acute and chronic inflammation.

The surgical interventions performed were key in the patient’s therapeutic approach, requiring a total of eight procedures over a period of 35 days, which included seven irrigations, one escharectomy, and two debridements designed to preserve viable tissue and promote wound healing; one placement of a VAC system, which helped maintain a controlled moist environment, facilitated exudate removal, and isolated the wound. Although the standard pressures for this system usually range from 100-125 mmHg, lower pressures are recommended in pediatric patients in order to prevent tissue damage. Additionally, three applications of hydrocolloid dressings were implemented with the purpose of enhancing granulation tissue formation and preventing contiguous infections, while also promoting surgical wound closure.

The final surgical intervention consisted of an Ollier-Thiersch dermoplasty, a split-thickness skin grafting technique involving the epidermis and part of the dermis. This method was chosen for its high graft take rate, attributed to its lower vascularization requirements, an important factor in this case, given the compromise of vascular beds caused by multiple debridement procedures, thus making the split-thickness graft the most suitable option [10].

Moreover, this type of graft enables coverage of extensive areas while also minimizing donor-site morbidity, an especially advantageous characteristic in this patient with an aggressive post-infectious condition, such as necrotizing fasciitis. Following this intervention, the disease was deemed resolved.

## Conclusions

The occurrence of necrotizing fasciitis is an uncommon but potentially life-threatening complication, in which the prognosis depends on early diagnosis and immediate treatment. Our case represents a prime example of the disease’s progression, clinical manifestations, and therapeutic approach described in the medical literature, achieving successful management with a multidisciplinary approach that combined pharmacotherapy, surgical interventions, and intensive care support. This report highlights the importance of having a trained medical team capable of recognizing and managing this condition, thereby reducing morbidity and mortality.

## References

[1] Schulz Schulz, S. A. (2025). Schulz SA: Necrotizing fasciitis: Practice essentials, pathophysiology, etiology. Medscape.

[2] Aranda JA, Velasco RF, Ibarra D, Herrera MG (2016). Manual de pediatría. Hospital infantil de México. Ciudad de México.

[3] Parra CP, Pérez ES, Patiño ME (2012). Actualización en fascitis necrotizante. Seminarios de la Fundación Española de Reumatología.

[4] Centers for Disease Control and Prevention. (2025, August 7 (2025). Centers for Disease Control and Prevention: Necrotizing Fasciitis. https://www.cdc.gov/group-a-strep/about/necrotizing-fasciitis.html.

[5] Wallace HA, Perera TB (2025). Necrotizing Fasciitis. National Library of Medicine.

[6] Longo D, Fauci A, Kasper D (2025). Harrison's Principles of Internal Medicine, 22nd Edition.

[7] Brunicardi FC, Andersen DK, Billiar TR (2020). Schwartz's Principles of Surgery, 11e.

[8] Salati SA (2022). Necrotizing fasciitis a review. Pol Przegl Chir.

[9] Andreassi A, Bilenchi R, Biagioli M, D'Aniello C (2005). Classification and pathophysiology of skin grafts. Clin Dermatol.

[10] (2025). Necrotizing Fasciitis (Flesh-Eating Bacteria). https://www.webmd.com/skin-problems-and-treatments/necrotizing-fasciitis-flesh-eating-bacteria.

